# A Qualitative Analysis of Patient Experiences Using Semaglutide 2.4 mg for Weight Loss

**DOI:** 10.1002/osp4.70085

**Published:** 2025-08-06

**Authors:** Eion Plenn, Dylan Amin, Jonathan Henry, Gabrielle Leavitt, Jason Walker, Taraneh Soleymani

**Affiliations:** ^1^ Penn State College of Medicine Hershey Pennsylvania USA; ^2^ St. George's University School of Medicine West Indies Grenada; ^3^ Cooper Medical School of Rowan University Camden New Jersey USA; ^4^ Lewis Katz School of Medicine Temple University Philadelphia Pennsylvania USA; ^5^ Geisinger Commonwealth School of Medicine Scranton Pennsylvania USA

**Keywords:** GLP‐1 receptor agonists, mental health, semaglutide 2.4 mg, weight stigma

## Abstract

**Introduction:**

Semaglutide 2.4 mg (Wegovy) is the first second‐generation anti‐obesity medication approved by the FDA for long‐term obesity treatment. Given the effectiveness of semaglutide 2.4 mg compared to first‐generation anti‐obesity medications and amid ongoing national shortages, understanding the real‐world experiences of individuals using semaglutide 2.4 mg is crucial. This qualitative study aims to explore the narratives of individuals using semaglutide 2.4 mg for weight management, as expressed in Reddit posts.

**Methods:**

One thousand posts from the r/WegovyWeightLoss subreddit between October 10, 2022, and October 6, 2023, were retrospectively analyzed. After excluding irrelevant posts, 660 posts were included in the analysis. A codebook was collaboratively developed and data was coded using qualitative software. Themes were finalized and reviewed.

**Results:**

Seven themes were identified from the analysis: medication efficacy (29.4% of coded posts), psychosocial impact of weight loss (22.8%), side effects management (13.3%), barriers to access (10.7%), lifestyle modification (10.2%), support and community (9.2%), and stigma surrounding use of semaglutide 2.4 mg for weight loss (4.4%).

**Conclusion:**

This qualitative analysis of Reddit posts provides valuable insights into the real‐world experiences of individuals using semaglutide 2.4 mg for weight management. By understanding these user experiences, healthcare providers can better tailor obesity treatment plans and enhance patient‐centered care for those using second‐generation anti‐obesity medications like semaglutide 2.4 mg.

## 1 Introduction

Among anti‐obesity medications, glucagon‐like peptide receptor agonists (GLP‐1RA) have garnered significant attention in both the public and medical community for their substantial weight loss results [1, 2, 3]. The first GLP‐1RA approved by the FDA for chronic management of obesity was a weekly subcutaneous injection containing semaglutide 2.4 mg. Clinical trials demonstrated that individuals receiving semaglutide 2.4 mg achieved 15%–17% average weight reduction that was sustained over 68 weeks [4]. Furthermore, semaglutide 2.4 mg demonstrated improvements in cardiometabolic parameters, including glycemic control and blood pressure, as well as in behavioral patterns, such as improved eating habits [5, 6, 7]. The treatment of obesity with semaglutide 2.4 mg presents challenges for both patients and providers, including managing side effects [8], insurance coverage issues [9], and frequent national shortages of the medication [10]. Additionally, individuals with obesity are disproportionately affected by societal stigma and often face bias from healthcare professionals [11, 12, 13, 14].

Despite clinical trials demonstrating the impressive weight loss outcomes from semaglutide 2.4 mg, patient experiences using the medication remain underexplored. Fortunately, semaglutide 2.4 mg and other GLP1‐RAs are extensively discussed across a range of online platforms [15], including the social media application Reddit. Reddit is a global social discussion platform with over 16 billion posts and comments, and more than 70 million new user interactions per day [16]. Over the past decade, there has been a surge in qualitative investigations using the perspective of Reddit users [17, 18, 19]. Reddit has become a resource for research studies due to its size, accessibility, and sense of anonymity [20], resulting in a large pool of data, especially on uncommon or stigmatized health concerns such as obesity. Conversations on Reddit regarding GLP‐1RAs and their impact on mental health, substance use, and addiction have already begun to be explored using qualitative research on this platform [21, 22, 23].

This study aimed to investigate patient experiences with semaglutide 2.4 mg (sold by Novo Nordisk as Wegovy for weight loss) by analyzing data from the subreddit r/WegovyWeightLoss. By gathering firsthand narratives, we sought to gain a deeper understanding of a patient perspectives and society's view on semaglutide 2.4 mg.

## 2 Materials and Methods

The shared experiences of semaglutide 2.4 mg users on the subreddit r/WegovyWeightLoss were explored. This subreddit is described as “an unofficial community for people who use or are interested in Wegovy or other GLP‐1RAs medications for weight loss.” At the time of this study (October 6, 2023), the subreddit had 25,820 subscribed users. This study was exempt from research ethics board approval as it relied on information in a public forum, meaning that the subjects of this research would have had no reasonable expectation of privacy.

### 2.1 Data Collection

APIFY was used to scrape the titles and body text of all posts from the subreddit r/WegovyWeightLoss on Oct 6, 2023 (*n* = 1000). Comments were excluded from the web scrape as they often diverged from the original topic of the post or were nonsubstantive (e.g., comments like “Thank you!” or “I agree.”). The dataset was then downloaded into Microsoft Excel and cleaned by (1) identifying the relevant data and (2) removing posts that were images (e.g., weight‐loss transformation photos), URLs, or blanks. After cleaning, a final dataset of 660 posts was obtained. Following a review of the posts, a preliminary codebook was applied. Posts from other GLP‐1RA‐related subreddits, including but not limited to r/Ozempic, r/OzempicForWeightLoss, and r/Semaglutide, were initially reviewed. However, it was determined that the 660 posts from r/WegovyWeightLoss were sufficient as thematic saturation had been reached. Thematic saturation was defined as the point at which no new codes or significant insights emerged from further analysis, indicating that the dataset adequately captured the diversity of user experiences with semaglutide 2.4 mg. We excluded r/Tirzepatide and r/Mounjaro as they focus on GLP‐1/GIP medications, which are different from the GLP‐1 receptor agonists used in this study.

To ensure continued relevance of the dataset, we revisited r/WegovyWeightLoss periodically throughout the study. These ongoing reviews confirmed that the core themes remained consistent over time. This supported our conclusion that thematic saturation had been achieved and that the dataset continued to reflect enduring patterns in user experiences with semaglutide.

### 2.2 Codebook Development and Data Analysis

A research team collaboratively developed a codebook using inductive coding based on themes identified through an iterative review of all posts, as described by Braun and Clark [24]. During the review of the initial 660 posts, the authors met multiple times to discuss emerging themes. For each potential theme, example posts were reviewed by the entire group to determine relevance. Areas of strong overlap were identified, and an initial set of codes was agreed upon. The finalized codebook was then validated by a physician specialized in obesity medicine (Table 1). Data were then analyzed according to the finalized codebook using MAXQDA software [25]. A total of 1114 coded references were reported in the software, as some posts were coded more than once. Coded references were summarized, and themes were developed by the research team.

**TABLE 1:** Example of inductive codebook for r/WegovyWeightLoss.

Code	Description
Weight loss	Any mention of weight loss secondary to semaglutide 2.4 mg
Physical health	Any mention of positive improvements to physical health, such as reductions in A1C, cholesterol, blood pressure, increases in physical activity, etc.
Reduction in food noise	Any mention of reductions in food noise or appetite suppression
Side effects	Any mention of negative side effects from semaglutide 2.4 mg
Health complications	Any mention of severe medical complications, hospitalizations, or surgeries
Shortages	Any mention of difficulties accessing semaglutide 2.4 mg due to national shortages
Insurance	Any mention of difficulties with insurance coverage or cost
Positive provider‐patient interactions	Any mention of positive provider‐patient interaction
Negative provider‐patient interactions	Any mention of negative provider‐patient interaction
Stigma	Any mention of bias or judgment from friends, family, or society, related to semaglutide 2.4 mg use
Support and community	Any mention of users relying on friends, family, or the Reddit community as a mode of support
Positive impact on mental health	Any mention of positive impacts on mental health secondary to semaglutide 2.4 mg, including increased self‐esteem, self‐confidence, feelings of hope, and perceived positive feedback from others
Negative impact on mental health	Any mention of negative impacts on mental health secondary to semaglutide 2.4 mg, including feelings of disappointment, ambivalence, fear, anxiety, and perceived negative feedback from others. This code includes new onset eating disorders, food aversions, and loss of food preferences
Lifestyle modification	Any mention of changes to lifestyle, including positive dietary adjustments and exercise regimen. This code also included changes to alcohol consumption and substance use
Stopping semaglutide	Any mention of stopping semaglutide 2.4 mg
Resources and advice	Any post that provided resources, motivation, or shared advice for using semaglutide 2.4 mg, not coded elsewhere

## 3 Results

Seven themes were identified from the analysis (Table 2): medication efficacy (29.4% of coded posts), psychosocial impact of weight loss (22.8%), side effects management (13.3%), barriers to access (10.7%), lifestyle modification (10.2%), support and community (9.2%), and stigma surrounding the use of semaglutide 2.4 mg for weight loss (4.4%).

**TABLE 2:** Frequency of themes with illustrative quotes from r/WegovyWeightLoss.

Themes	Number of coded references (*n* = 1114)	%	Codes	Illustrative quote
Medication efficacy	328	29.4	Weight loss	“I am under 300 lbs. for the first time in 5 years, this morning.”
			Physical health	“I'm feeling lighter. More physically fit, too. Busting through my daily walk and feeling like I could keep going for another mile or more.”
			Reduction in food noise	“The difference is I've shut off the noise in my brain and not constantly focused on my next meal.”
Psychosocial impact of weight loss	254	22.8	Positive impact on mental health	“This medicine has given me back the control I needed in my life.”
			Negative impact on mental health	“I felt incredibly discouraged when I saw so many people posting about losing on doses where I lost nothing.”
Managing side effects	148	13.3	Side effects	“The severe nausea I had when first starting has improved a lot.”
			Health complications	“Did a month of 0.25 and skipped up to 1 because it's all my doctor and I could find in stock. 2 weeks later and I have pancreatitis. I'm the sickest I have ever been.”
			Stopping semaglutide 2.4 mg	“I think I'm going to stop Wegovy. Took my second dose yesterday and had the same reaction, if not worse. I did not eat bad or drink alcohol. Been up since 3 p.m. yesterday vomiting and diarrhea.”
Barriers to access	119	10.7	Shortages	“My new PCP prescribed Wegovy 1 mg on July 26th; however, due to the shortage of the initial doses, I was unable to fill the prescription.”
			Insurance	“Paying out of pocket for Wegovy is unsustainable.”
Lifestyle modification	114	10.2	Lifestyle modification	“I'm eating way less, with lots of whole foods, exercising and feeling wonderful about myself!”
Support and community	102	9.2	Support and community	“I have found the majority of posts and comments to be so inspiring and helpful.”
			Resources and advice	“Everyone's body and journey will be different…commit to being in it for the long haul.”
			Positive provider‐patient interaction	“For once, I have a doctor who actually understands my struggle and even confided in me that she is also taking Wegovy. She has helped me to not get discouraged and it's so nice to feel gaslit by a doctor!”
Stigma surrounding weight loss medication	49	4.4	Stigma	“Someone I told insinuated that I was cheating by taking Wegovy even though she knew full well how much work I put in every single day for so many years.”
			Negative provider‐patient interaction	“I've seen doctor after doctor and they all say the same thing: ‘you need to eat less and exercise more and keep a food journal.’ Like, wow, I've been overweight my whole life and have never heard/thought of that before. Thanks doc.”

### 3.1 Theme 1: Medication Efficacy

Users consistently reported slow but sustainable weight loss with semaglutide 2.4 mg, with some achieving milestones such as reaching healthy BMI ranges or losing substantial amounts of weight. Users experienced a wide range of health improvements, including blood sugar regulation, diabetes reversal, and significant reductions in cholesterol and blood pressure levels. Users reported a significant reduction in “food noise,” characterized by decreased food‐related intrusive thoughts and maladaptive eating behaviors. Weekly injections of semaglutide 2.4 mg enabled users to regain control over their eating habits, leading to a cessation or reduction in binge‐eating patterns, compulsive eating, and emotional eating.

I've had binge eating disorder since high school. My symptoms are gone. Desire to snack? Gone. Food obsession? Gone. I'm hopeful for the first time in ages.

### 3.2 Theme 2: Psychosocial Impact of Weight Loss

Users reported a range of positive experiences as a result of their weight loss journey with semaglutide 2.4 mg. These included feelings of increased self‐confidence, self‐esteem, hope, and happiness. Several users reported improvements in multiple mental health conditions, including anxiety, depression, and compulsive behaviors (e.g., compulsive shopping) since starting semaglutide 2.4 mg. Non‐scale victories, such as fitting into smaller clothes and experiencing physical changes, contributed to enhanced self‐esteem and feelings of accomplishment.

Despite the positive experiences, some users expressed feelings of discouragement, frustration, and anxiety, particularly when progress was slow or inconsistent. Some users struggled to reconcile with their new appearance, experiencing feelings of disconnection and discomfort:

I’m feeling so disconnected from my appearance. It’s like every time a picture is taken of me I see a different person…I’m so accustomed to it being tied to a body I feel so terrible about

Perceived negative feedback from others, such as unwelcome attention or comments on weight loss, contributed to feelings of discomfort and self‐consciousness. For example, some users reported unwanted attention from the opposite sex since losing weight. Additionally, disordered eating patterns and food aversions emerged as significant challenges for some users, further exacerbating feelings of anxiety and loss around food.

### 3.3 Theme 3: Managing Side Effects

Users discussed experiencing a variety of side effects with semaglutide 2.4 mg, including gastrointestinal discomfort, nausea, diarrhea, constipation, headaches, and fatigue:

The side effects are no joke. For me, I take my shot on a Wednesday and by Thursday night, the nausea and explosive diarrhea starts and lasts a solid two days.

Additionally, some users described significant health complications attributed to semaglutide 2.4 mg use, such as episodes of severe constipation leading to emergency room visits, gallbladder issues, abdominal pain, appendicitis, and pancreatitis. Users emphasized the importance of closely monitoring symptoms and promptly seeking medical attention if any unusual or concerning side effects occur.

### 3.4 Theme 4: Barriers to Access

Users discussed difficulties in accessing semaglutide 2.4 mg due to shortages, which led to anxiety and frustration about filling their prescriptions. Some users encountered waiting lists for the medication, while others received notices from their insurance discontinuing coverage, exacerbating feelings of demoralization and hopelessness. Financial barriers were a significant concern, with users expressing concerns about the high cost of semaglutide 2.4 mg and lack of insurance coverage. Some users shared their experiences of paying out‐of‐pocket for semaglutide 2.4 mg, describing it as unsustainable or unaffordable for many individuals. Concerns about insurance prior authorization expiring and the uncertain nature of access to semaglutide 2.4 mg due to insurance dynamics were prevalent among users, leading to ongoing anxieties about medication access. In response, users offered advice and support to navigate barriers to access, such as researching copay assistance programs, comparing prices at different pharmacies, and advocating for insurance coverage through appeals. When users exhausted their options, some turned to acquire semaglutide 2.4 mg from compounding pharmacies:

I went to my primary care doctor yesterday. I had sent her multiple messages through the portal they have about my troubles finding a prescription for 0.5 mg. I asked she send it to Amazon, and got no response. She is referring me to a weight management place where they can give me compounds.

### 3.5 Theme 5: Lifestyle Modification

Users described adopting various dietary modifications, such as prioritizing high‐protein and low‐carbohydrate meals and focusing on balanced, nutrient‐dense options. They emphasized the importance of portion control, mindful eating, and avoiding trigger foods to manage discomfort or overeating. In terms of exercise, users reported an increase in physical activity, particularly in strength training routines involving bodyweight exercises, dumbbells, and swimming.

Users also reported significant changes in alcohol and substance consumption since starting semaglutide 2.4 mg, with many experiencing a decrease in alcohol intake. Others shared their experiences of prior alcohol or substance addiction and credited semaglutide 2.4 mg for aiding their newfound sobriety, highlighting the medication's potential impact on addictive behaviors.

Alcohol cravings no longer exist. Before Wegovy, I would want a beer or glass of wine every day…but I don't even think about it anymore.

### 3.6 Theme 6: Support and Community

Users frequently expressed gratitude for the subreddit community as a supportive and safe space for sharing experiences, seeking advice, and finding motivation during their semaglutide 2.4 mg journeys.

We love you. We understand the struggle. We support you. Fight the good fight, stay the course, hold the line, and press on.

Users also recounted uplifting encounters with healthcare providers who offered support, encouragement, and understanding.

### 3.7 Theme 7: Stigma Surrounding Weight Loss Medication

Users recounted instances of judgmental remarks and stigma from acquaintances, friends, and family members regarding their use of semaglutide 2.4 mg. This included encounters where natural methods of weight loss were valorized over medication‐assisted approaches, leading to feelings of shame and embarrassment. Users also shared experiences of negative interactions with healthcare providers, including feeling dismissed, misunderstood, or stigmatized due to their weight or their request for semaglutide 2.4 mg. Some recounted encounters where doctors offered simplistic solutions such as exercise and eat less,” without understanding the complexities of obesity treatment or considering the individual's specific needs.

I've hired a nutritionist. I've hired a personal trainer. I've seen doctor after doctor and they all say the same thing 'you need to eat less and exercise more and keep a food journal.'

## 4 Discussion

The analysis of data collected from the subreddit “r/WegovyWeightLoss” provides valuable insights into the experiences of individuals using semaglutide 2.4 mg for weight management. This study identified several key themes that shed light on diverse aspects of the semaglutide 2.4 mg experience, including medication efficacy, psychosocial impact of weight loss, managing side effects, barriers to medication access, lifestyle modification, support and community, and stigma surrounding weight loss medication.

One prominent finding from our analysis highlights the effectiveness of semaglutide 2.4 mg in facilitating substantial weight loss among users of the medication. Our findings are consistent with the current literature surrounding semaglutide's effectiveness in achieving greater weight loss, improving or resolving obesity‐related comorbidities, and reducing food noise and disordered eating patterns [6, 7]. Users have also noted alterations in alcohol consumption and substance use following the initiation of semaglutide 2.4 mg, contributing to the existing body of literature supporting GLP‐1RA as a potential treatment approach for addiction [21, 23, 26, 27]. However, alongside the positive outcomes, users also discussed experiencing various side effects associated with semaglutide 2.4 mg use. Gastrointestinal discomfort, headaches, and fatigue were commonly reported side effects, prompting users to develop strategies for managing these symptoms [8]. Importantly, this study identified instances of significant health complications attributed to semaglutide 2.4 mg, underscoring the need for close monitoring and prompt medical attention for individuals experiencing adverse reactions [3].

This study also explored the psychosocial impact of significant weight loss associated with semaglutide 2.4 mg use, revealing a complex interplay between changes in self‐confidence, self‐esteem, mental health, and interpersonal relationships. These findings align with existing literature, particularly studies examining the psychological impact of significant weight reduction achieved via bariatric surgery [28]. For example, a review by Sarwer and Heinberg provided a more contemporary overview of the psychosocial aspects of obesity and bariatric surgery, which aligned with the findings of this study. Similar to the bariatric surgery patients, semaglutide 2.4 mg users experienced improvements in body image and appearance, while for some, weight loss led to psychologically distressing outcomes including worsening self‐image. It was also observed that when individuals' weight loss expectation from semaglutide 2.4 mg did not match the actual weight loss, users reported feelings of frustration and disappointment [29]. This finding adds to the existing pool of literature demonstrating significant disparities between patients' expectations and what is recommended by health care providers [30, 31], emphasizing the importance of educating patients on the range of potential weight loss outcomes and treatment response heterogeneity.

Some users reported strains in marital relationships and unwanted attention from males following weight loss, echoing observations from past research [32]. Consistent with a qualitative study on weight stigma after bariatric surgery, our research revealed discomfort among some users when others commented on their weight loss, with many expressing displeasure at the unwanted attention [33].

A noteworthy discovery was the emergence of feelings of anxiety and loss related to food aversions after initiating semaglutide 2.4 mg. While these findings are sourced from Reddit discourse, they raise awareness of the potential for anti‐obesity medications to worsen or lead to new disordered eating patterns and subsequent psychiatric comorbidities. Overall, these findings emphasize the importance of monitoring and providing behavioral health support as patients undergo transformational weight loss.

Access to semaglutide 2.4 mg emerged as a significant barrier for many users, with shortages [10] and insurance coverage issues hindering medication availability and equity [9]. These obstacles have led some patients to obtain semaglutide 2.4 mg from compounding pharmacies, which prepare their own medications to meet patients' needs independent of FDA approval. The FDA along with multiple professional societies including The Obesity Medicine Association and The Obesity Society has advised against the potential dangers that compounded peptides pose [34, 35]. As described by Gudeman et al., compounding pharmacies may increase the risk of medical errors due to lack of standardized quality control measures, particularly when producing drugs on a large scale [36]. Currently, there are three reports in the literature of harmful administration of semaglutide 2.4 mg obtained from compounding pharmacies [36]. More research should be conducted on the role of compounding pharmacies in accessing semaglutide 2.4 mg, with a particular focus on evaluating the risks and benefits in obesity management.

This study highlighted the critical role of community support in achieving weight loss goals. This theme was consistent with other qualitative studies of Reddit [19, 20], where users consistently expressed appreciation for the subreddit community as a secure and supportive platform for sharing experiences, seeking advice, and finding motivation. Moreover, Dicker et al. found that positive interpersonal relationships with healthcare providers were associated with increased patient motivation to lose weight [37]. Similarly, this study revealed the presence of this theme, as users highlighted the significance of supportive healthcare providers who offered encouragement and understanding for the management of obesity.

Finally, the theme of stigma surrounding weight loss medication use emerged prominently in this study, with users sharing instances of encountering judgmental remarks and negative experiences with healthcare providers. Despite being reported less frequently in our cohort (4.4% of posts), it remains an important aspect to highlight as it reflects the pervasive nature of weight bias and stigma [11]. These findings underscore the critical need to address stigma within healthcare settings.

### 4.1 Limitations and Strengths

Several limitations were present in this study. Firstly, a lack of user demographic information was noted, as Reddit community users are identified solely by their usernames. Additionally, information regarding the duration of semaglutide 2.4 mg use for all community users could not be obtained, which impacts the generalizability of the findings. Moreover, the study focused solely on data collected from Reddit, overlooking potential insights from other social media platforms such as TikTok and Instagram. Although other Reddit communities focused on semaglutide 2.4 mg were examined and thematic saturation was deemed to have been reached in the r/WegovyWeightLoss subreddit, it remains possible that relevant posts and comments were excluded from the search as Reddit is a dynamic platform. Furthermore, the decision to rely solely on data from a single subreddit constitutes a limitation in itself, as different subreddits may have distinct “cultures” or may collectively trend toward certain viewpoints more strongly than others. Despite these limitations, the study included an extensive sample size, enhancing the robustness and reliability of the findings and likely achieving a representative sample of themes and perspectives related to semaglutide 2.4 mg use.

## 5 Conclusion

Overall, this study provides valuable insights into the experiences of individuals using semaglutide 2.4 mg for weight management, highlighting the multifaceted nature of the semaglutide 2.4 mg journey and emphasizing the importance of addressing systemic barriers, psychosocial concerns, and stigma to support individuals in achieving their health goals. This analysis of posts on the subreddit r/WegovyWeightLoss has illuminated several critical insights into the experiences of individuals using semaglutide 2.4 mg for weight loss. The themes identified encompass a broad spectrum of challenges and successes encountered by users, ranging from medication efficacy to psychosocial impact and barriers to access. These findings may inform the development of strategies to address the information needs of patients using semaglutide 2.4 mg, with the ultimate goal of creating a patient‐centered approach for optimizing the use of second‐generation anti‐obesity medications in obesity management. Further research is needed to explore the long‐term effects and outcomes of semaglutide 2.4 mg use as well as the broader implications for obesity treatment and public health policy.

## Conflicts of Interest

The authors E.P., D.A., J.H., G.L., and J.W. declare that they have no conflicts of interest to disclose regarding the publication of this work. TS reported receiving a speaking fee from Novo Nordisk and an honorarium from the American Board of Obesity Medicine.

## Data Availability

The data that support the findings of this study are available from the corresponding author upon reasonable request.
